# Age-Related Changes of the Synucleins Profile in the Mouse Retina

**DOI:** 10.3390/biom13010180

**Published:** 2023-01-15

**Authors:** Sarah Batista Dias, Luísa de Lemos, Luís Sousa, Diogo B. Bitoque, Gabriela Araújo Silva, Miguel C. Seabra, Sandra Tenreiro

**Affiliations:** 1 iNOVA4Health, NOVA Medical School|Faculdade de Ciências Médicas, NMS|FCM, Universidade Nova de Lisboa, 1169-056 Lisboa, Portugal; 2 UCL Institute of Ophthalmology, London EC1V 9EL, UK

**Keywords:** synuclein, retina, aging, degeneration

## Abstract

Alpha-synuclein (aSyn) plays a central role in Parkinson’s disease (PD) and has been extensively studied in the brain. This protein is part of the synuclein family, which is also composed of beta-synuclein (bSyn) and gamma-synuclein (gSyn). In addition to its neurotoxic role, synucleins have important functions in the nervous system, modulating synaptic transmission. Synucleins are expressed in the retina, but they have been poorly characterized. However, there is evidence that they are important for visual function and that they can play a role in retinal degeneration. This study aimed to profile synucleins in the retina of naturally aged mice and to correlate their patterns with specific retinal cells. With aging, we observed a decrease in the thickness of specific retinal layers, accompanied by an increase in glial reactivity. Moreover, the aSyn levels decreased, whereas bSyn increased with aging. The colocalization of both proteins was decreased in the inner plexiform layer (IPL) of the aged retina. gSyn presented an age-related decrease at the inner nuclear layer but was not significantly changed in the ganglion cell layer. The synaptic marker synaptophysin was shown to be preferentially colocalized with aSyn in the IPL with aging. At the same time, aSyn was found to exist at the presynaptic endings of bipolar cells and was affected by aging. Overall, this study suggests that physiological aging can be responsible for changes in the retinal tissue, implicating functional alterations that could affect synuclein family function.

## 1. Introduction

Synucleins are a family of intrinsically unstructured proteins, including alpha- (aSyn), beta- (bSyn) and gamma-synuclein (gSyn). aSyn is the most studied member of this family due to its central role in Parkinson’s disease (PD). Point mutations, duplications and triplications of the aSyn encoding gene (*SNCA*) are associated with rare familial cases of PD. aSyn is also the major component of Lewy Bodies (LBs), the aggregates that are hallmarks of PD and other neurodegenerative disorders known as synucleinopathies. The toxic role of aSyn is by far more understood than its physiological role due to the availability of cellular and animal models of PD where aSyn toxicity has been extensively studied [1]. Degradation pathways and autophagy, mitochondria function and vesicular trafficking are severely affected by aSyn oligomerization/aggregation, altogether leading to increased oxidative stress and impaired proteostasis [1,2]. In turn, macroautophagy as well as several chaperone-mediated protein quality control mechanisms are known to decrease efficacy with aging [3,4], and its correct functioning is essential for aSyn degradation and proteostasis [1]. Impairment of these pathways increases aSyn aggregation [1,5]. bSyn and gSyn may also cause neurotoxicity. Namely, mutations in bSyn are associated with rare cases of dementia with LBs (DLBs) [6]. In yeast cells, bSyn overexpression results in the formation of inclusions, vesicular trafficking impairment, oxidative stress and ultimately cytotoxicity and cell death [7]. In addition, bSyn has been found to be neurotoxic in primary neuronal cultures and in the rat substantia nigra (SN) [8]. gSyn is associated with neurodegeneration in glaucoma [9]. Inclusions of gSyn have also been reported in the brains of some patients with PD, DLB and Hallervorden-Spatz syndrome [10,11]. Transgenic mice overexpressing gSyn develop severe age-dependent neuropathology and motor deficits and die prematurely, presenting gSyn aggregation in cytoplasmic and axonal lesions, features that resemble amyotrophic lateral sclerosis (ALS) in humans [12,13]. Indeed, aggregated gSyn has been found in the motor neurons of some ALS patients [12].

The functional role of aSyn and its homologs is far from being understood. Synucleins seem involved in synaptic vesicle endocytosis and exocytosis; aSyn interacts with Rab GTPases (Rab3a, Rab5 and Rab8), important regulators of membrane trafficking, namely budding, tethering, docking and fusion of vesicles, pointing to a role in vesicle recycling [14]. aSyn is also required for assembly of the soluble N-ethylmaleimide sensitive fusion attachment protein receptor (SNARE) complex, which is crucial in maintaining neurotransmitter release machinery at the synapse [15]. aSyn has also been found to regulate presynaptic terminal size and synaptic vesicle distribution [16] and to help dilate the fusion pore, which forms transiently during vesicle exocytosis, promoting the release of neurotransmitters, with particular relevance for dopamine release [17]. Several studies using single, double and triple knockout mice of synuclein family members addressed how synucleins can functionally compensate each other [18,19,20,21,22,23,24,25,26]. Overall, data point to just some degree of redundancy, with not complete functional compensation by the different family members, depending on the cellular process being addressed, the brain region under analysis or the age of the animal model used in the different studies. The important idea emerging from these studies is that although synucleins are not essential components of the basic machinery for neurotransmitter release, they seem to contribute to the long-term maintenance of presynaptic function, and this role is of special relevance in aging [18,20,27,28]. Additionally, it has been shown that old age aggravates the propagation of aggregated aSyn in transgenic and wild-type animal models (reviewed in [29]).

Furthermore, regarding their synergistic/additive toxic role, there are contradictory results. Although the toxic role of bSyn has been reported in rare cases of DLBs [6] and in cellular models [7,8], several studies have pointed to a protective role of bSyn on aSyn toxicity by reducing its seeding and toxicity [30,31,32]. Interestingly, we previously demonstrated that aSyn and bSyn can form heterodimers in yeast and mammalian cells [7].

Synucleins are highly expressed in the neuroretina and retinal pigmented epithelium [33,34], but their physiological and/or toxic role in this tissue is very poorly characterized. However, evidence shows that these proteins may play important roles in visual function and are mainly affected by aging, as the triple knockout (KO) mice display age-dependent retinal dysfunction and blindness, even if they only present mild alterations in synaptic structure and transmission, as well as diminished survival [28]. Additionally, retinal synucleinopathy could be responsible in part for visual impairment and loss as observed in PD, one of the nonmotor symptoms these patients present [35,36]. Retinas from PD patients’ show electrophysiological and morphological alterations revealed by electroretinogram (ERG) and coherence tomography (OCT) evaluations [35]. At the molecular level, aggregated aSyn is mainly phosphorylated at Serine 129 (pS129) [37], and pS129 aSyn was detected in the retina of PD patients [38,39]. This is the main post-translational modification found in aggregated aSyn in the Lewy Bodies in the brains of PD patients [37], and it is used as a marker of aggregation. Moreover, in PD retinas, aggregated aSyn has been found to be located in the inner plexiform layer (IPL) (which contains dopaminergic amacrine cells) and in retinal ganglion cells (RGCs) (prime targets for modulation by dopamine) [40]. aSyn distribution has been found to be altered in retinal neurons in DLB [41], and age-related aSyn inclusions have been observed in human retinas [42]. gSyn inclusions have also been observed in the outer plexiform layer (OPL) in Alzheimer’s disease (AD) [33] and in the IPL of aged degenerating retinas with glaucoma [43]. Importantly, gSyn was also associated with neurodegeneration in glaucoma [9]. Altered gene expression, protein levels and neuronal distribution of bSyn have also been found in aged monkey retinas [44]. However, the available studies about retinal synucleinopathy in PD are basically descriptive, performed with a small sample of patients [38,40,45,46], and lack correlation between observations and disease progression.

Transgenic mouse models of aSyn overexpression or the use of adeno-associated viral (AAV) vectors to overexpress human aSyn in the mice’s inner retina were explored to clarify the aSyn toxic role in the retina [33,47,48]. These models have limitations, as aSyn overexpression can occur in retinal populations where naturally the protein is not expressed, and they show an accumulation of aSyn in specific retinal cells depending on the promoter used for expression. However, they confirmed the accumulation of pS129 aSyn in the retina [33,47,48].

Here, we characterized synuclein distribution in the retina of naturally aged wild-type (WT) mice up to 24 months of age and correlated it with specific retinal cell types by immunohistochemistry (IHC). As expected, we observed a significant decrease in the retinal thickness of several layers with aging, namely on the outer nuclear layer (ONL) cell nuclei number, in the outer segments (OS) layer thickness, and in the number of Brn3a positive ganglion cells. Additionally, neuroinflammation and glial reactivity were shown to be increased in the retina of aged mice. Based on IHC, aSyn total levels decreased with aging, while the bSyn levels were higher in the retinas of 24-month-old mice when compared with 6-month-old mice. However, aSyn and bSyn colocalization in the IPL decreased with aging. Regarding gSyn, whose main localization is in the ganglion cell layer (GCL) and inner nuclear layer (INL) cell bodies, its levels decreased with aging in the INL, while no significant alterations occurred in the GCL.

## 2. Materials and Methods

### 2.1. Animals

C57BL/6J WT mice were obtained from Jackson Laboratory (Bar Harbor, Maine), and the colony was kept at the NMS animal house in SPF conditions. Mice were housed in conventional cages in a 12 h dark/12 h light cycle at an average temperature of 21 °C with food and water ad libitum. Male mice were euthanized at 6, 9, 12, 18 and 24 months of age. All procedures were conducted in accordance with the Portuguese National Authority (DGAV) and Association for Research in Vision and Ophthalmology (ARVO) guidelines and following Portuguese legislation (DL113/2013) and European Directives (2010/63/EU) for the use of animals in the laboratory.

### 2.2. Sample Processing and Cryostat Sectioning

After humane sacrifice of the mice, both eyes of each animal were enucleated and washed twice in phosphate buffer saline 1X (PBS) and afterwards fixed in 4% paraformaldehyde (PFA) for 30 min under agitation. After fixation, the eyes were submitted to sucrose gradients of 10% and 20%, for 1 h each, and stored in 30% sucrose at 4 °C for 48 h prior to cryopreservation. Then, the eyes were embedded in Optimum Cutting Temperature medium (OCT) in a plastic mold and frozen at −80 °C.

Serial sections of 12 µm of thickness were performed using Leica Cryostat CM3050 S. Cryostat sections were stored at −20 °C until IHC characterization.

### 2.3. Immunohistochemistry

Cryostat sections stored at −20 °C were defrosted at room temperature (RT) for 2 h and hydrated afterwards in PBS for 30 min. Then, eye cryosections were permeabilized using 0.25% Triton X-100 in PBS (0.25% PBS-T) with three washes of 15 min each. For blocking, slides were incubated for 1 h at RT with blocking solution composed of 1% bovine serum albumin (BSA) and 3% Donkey serum in 0.25% PBS-T. Primary antibodies were incubated at specific concentrations in 0.25% PBS-T overnight at 4 °C. Then, samples were washed three times in 0.25% PBS-T and incubated with the respective secondary antibodies diluted at 1:1000 in 0.25% PBS-T for 1 h at RT. Slides were incubated for 10 min with 4′,6-diamidino-2-phenylindole (DAPI). After three washes, the slides were mounted using Mowiol mounting medium.

The following primary antibodies were used in the experiments: rabbit monoclonal anti-Vimentin (Cell signaling, Cat#5741, 1:400), mouse monoclonal anti-GFAP (Santa Cruz, Cat#sc-33673, 1:200), rabbit polyclonal anti-Iba 1 (Wako, 019-19741), mouse monoclonal anti-Rhodopsin [1D4] (Abcam, Cat#ab5417, 1:100), goat polyclonal anti-Brn3a (Santa Cruz, Cat#sc-31984, 1:100), mouse monoclonal anti-aSyn (BD, Cat#610787, 1:200), rabbit polyclonal anti-aSyn (Cell Signaling, Cat#2628S, 1:200), rabbit polyclonal anti-bSyn (Abcam, Cat#ab76111, 1:200), rabbit polyclonal anti-gSyn (Abcam, Cat#ab55424, 1:200); mouse monoclonal anti-PKCα (Santa Cruz, Cat#sc-8393, 1:200), mouse monoclonal anti-Synaptophysin (Sigma, Cat#S5768, 1:200), mouse monoclonal anti-Calbindin (Sigma, Cat#9848, 1:200). The secondary antibodies used were donkey anti-rabbit Alexa Fluor 488 Invitrogen (Cat#A-21206, Thermo Fisher Scientific, Waltham, MA, USA), donkey anti-mouse Alexa Fluor 568 Invitrogen (Cat#A-10037, Thermo Fisher Scientific, Waltham, MA, USA), donkey anti-goat Alexa Fluor 647 Invitrogen (Cat#A-21447, Thermo Fisher Scientific, Waltham, MA, USA), and donkey anti-mouse Alexa Fluor 647 Invitrogen (Cat#A-31571, Thermo Fisher Scientific, Waltham, MA, USA).

### 2.4. Confocal Microscopy and Fluorescence Image Analysis

Confocal z-stacks of immunostained sections were visualized on a Zeiss LSM710 confocal microscope (Zeiss, Germany) using a 40× water objective and 488 nm, 543 nm or 633 nm lasers.

Confocal images were processed using Image J—Fiji Software (Version 2.0.0., NIH). A minimum of 10 stacks were used for each maximum intensity Z-projection. Fluorescence intensity quantification was performed based on the mean of four different retinal sections of each animal, two from the peripheral region of the retina and two from the central region.

The cells were counted using the Image J cell counter tool. For that, we first determined the specific area and counted the cells of interest. Next, we normalized the total number of cells in that region. For fluorescent intensity analysis, the correspondent channel was selected, and the raw intensity was measured. Colocalization was calculated using the Manders’ coefficient.

### 2.5. Statistical Analysis

Statistical analysis was performed using GraphPad Prism software version 8.4.3. The results are presented as mean ± standard deviation (SD). All analyses were performed using a one-way non-parametric ANOVA test with Tukey’s multiple comparisons significance evaluation. Changes were considered significant at * *p* < 0.05; ** *p* < 0.01; *** *p* < 0.001; and **** *p* < 0.0001.

## 3. Results

### 3.1. Age-Related Retinal Neurodegeneration

**Figure 1:**
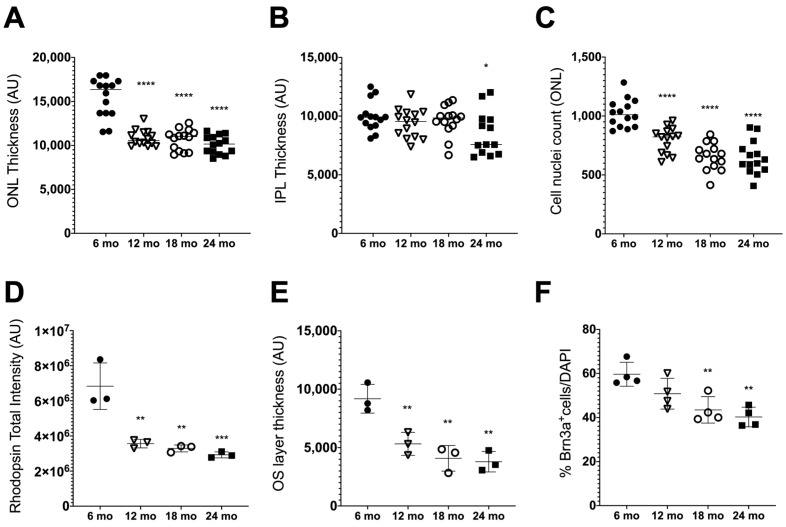
Evaluation of retinal layers in old mice. (**A**) Retinal thickness quantification of the outer nuclear layer (ONL) and (**B**) inner plexiform layer (IPL). (**C**) DAPI nuclei quantification of the ONL per field of view from mice at the indicated age (*n* = 14). (**D**) Quantification of the total intensity of the outer segment (OS) layer by immunofluorescence staining with Rhodopsin 1D4 in retinal sections. (**E**) Thickness measurements of the OS layer based on Rhodopsin 1D4 staining in retinal sections (*n* = 3). (**F**) Quantification of Brn3a-positive retinal ganglion cells (RGC) assessed by Brn3a immunofluorescence on retinal sections (*n* = 4). All quantification data are presented as mean ± SD. Mice of 12 (∇), 18 (○) and 24 months old (mo) (■) were compared to 6 mo (●) mice using an ordinary one-way ANOVA multiple comparison test (* *p* < 0.05, ** *p* < 0.01, *** *p* < 0.001 and **** *p* < 0.0001).

One of the most studied parameters for analyzing retinal degeneration is the decline of specific neuronal populations accompanied by functional deterioration. We first characterized the alterations in the thickness of each retinal layer of aged mice. The areas of IPL, INL, OPL and ONL in 6-, 12-, 18- and 24-month-old WT mice were measured based on nuclear DAPI staining of the retina assessed by confocal imaging (Figure 1 and Supplementary Figure S1). The results obtained showed that for the ONL, the area size decreased drastically in 12-, 18- and 24-month-old mice when compared to 6-month-old mice (control) and remained invariable for the older ages (Figure 1A). Regarding the IPL, this layer was thinner when comparing 24-month-old mice to the control, although for the other ages, it was not significantly affected (Figure 1B). The INL and OPL did not present significant thickness alterations with aging (results not shown). Since cell density and the number of cells are commonly used to evaluate age-related structural changes in retinal layers, we determined whether the thinning of the ONL was due to increased cellular density or cell loss. For that, we counted the number of DAPI-positive cell nuclei present in the ONL, and as expected, there was a significant decrease in the number of DAPI-positive cells in this layer in 12-, 18- and 24-month-old mice when compared to the control, pointing to retinal degeneration (Figure 1C).

Photoreceptor cells, especially rods, are known to be the most susceptible to damage and are among the first neurons to suffer functional decline and ultimately die in both mice and humans that are normally aging [49,50,51]. Here, to evaluate whether there were significative changes in these structures with aging, we used the Rhodopsin 1D4 antibody on retinal sections to identify the rod outer segments (OS), which correspond to the photosensitive compartment of photoreceptor cells, composed of rhodopsin disks (Figure 1D). The immunofluorescence analysis showed a clear decrease in the OS layer with age, with the disks less homogeneously distributed throughout the retinal section (Supplementary Figure S1). The quantification of the Rhodopsin total intensity signal showed a significative decrease in OS of older animals when compared with 6-month-old mice (Figure 1D). This decline in the Rhodopsin signal is in accordance with the significant reduction observed in OS area quantification (Figure 1E).

RGCs are also well known as being affected by aging [52,53]. We evaluated the loss of the RGC population with aging through immunofluorescence analysis of retinal sections of its specific marker, Brn3a, a commonly used marker for RGCs in mice and rat models [54], normalized by DAPI nuclei staining of this layer (Figure 1E, Supplementary Figure S1). A significant decrease in the number of Brn3a-positive cells was observed when comparing 18- and 24-month-old animals with the control. In retinas from 24-month-old mice, RGCs were becoming scarcer, suggesting cell degeneration (Figure 1E, Supplementary Figure S1).

Overall, we concluded that natural aging, followed until 24 months of age, reproduced the expected features of aging in the retina [53].

### 3.2. Age-Related Retinal Neuroinflamation

Inflammation is one important, established hallmark of aging [3,4]. Inflammation also has a known key role in PD [55]. Accordingly, we then evaluated neuroinflammation in the natural aging mice.

In the vertebrate retina, three basic glial cell types can be found: Müller cells, astrocytes, and microglia. The Müller cells are radial cells through the retinal tissue and have some of the functional attributes of astrocytes, reacting similarly to a variety of ocular injuries [56]. Müller cell reactivity is associated with an increased expression of vimentin or glial fibrillary acidic protein (GFAP) (or both), cell proliferation, and growth [57]. Astrocytes are closely associated with the nerve fiber layer and its reactivity hallmarks include increased thickness of cellular processes and increased intermediate filament protein expression [57].

**Figure 2:**
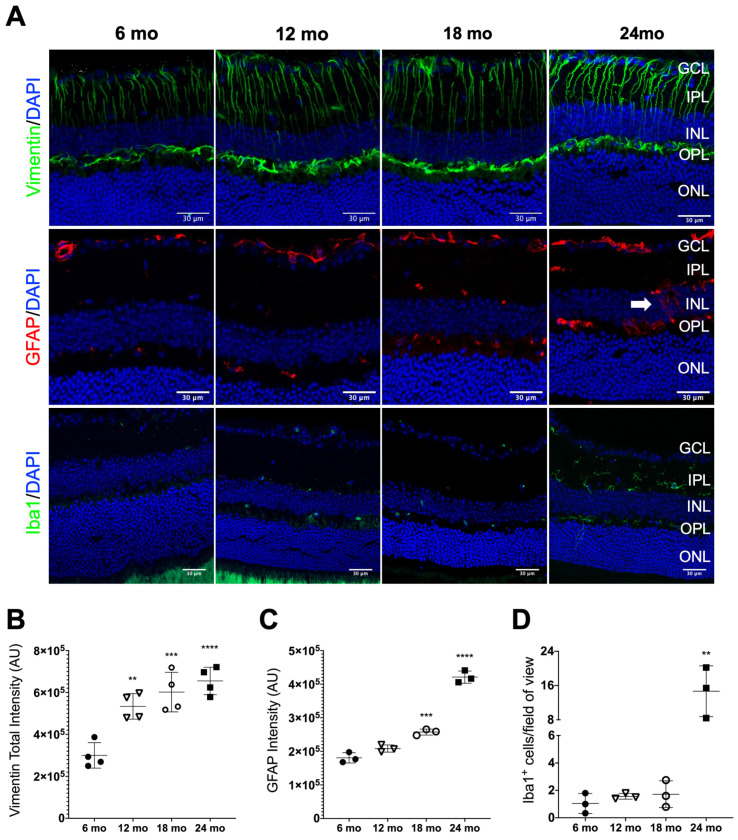
Age-related alterations in glial reactivity assessed by Vimentin, GFAP and Iba1 distribution profile in mice retinas. (**A**) Representative immunofluorescence staining for Müller glia (Vimentin), astrocytes (GFAP) and microglia (Iba1) counterstained with DAPI on retinal sections of mice at the indicated ages. In 24-month-old (mo) mice, GFAP immunoreactivity is prolonged throughout the INL indicated by the white arrow. (**B**) Quantification of vimentin fluorescence intensity throughout the retina for each age (*n* = 4). (**C**) Quantification of GFAP fluorescence intensity throughout the retina for each age (*n* = 3). Fluorescence intensity analysis was performed using approximately 4 representative images of the central region of the retina from each animal per age group. Values are represented by mean ± SD. Mice of 12 (∇), 18 (○) and 24 mo (■) were compared to 6 mo (●) mice using an ordinary one-way ANOVA multiple comparison test (** *p* < 0.01, *** *p* < 0.001 and **** *p* < 0.0001). Scale bar = 30 mm. (**D**) Quantification of Iba1^+^ cells detected throughout the retina for each age (*n* = 3). Microglia cell counts were performed per field of view of 5 representative images of each group. Values are represented by mean ± SD. Mice of 6, 12 and 18 mo were compared to 24 mo mice using an ordinary one-way ANOVA multiple comparison test (** *p* < 0.01).

We evaluated the levels of intermediate filament proteins vimentin and GFAP in normally aging mice. Immunofluorescence analysis showed vimentin fibers extending from the GCL throughout the IPL and INL, reaching the OPL (Figure 2A). With aging, this intermediate filament protein was clearly increasing, which was observed in the representative confocal images, especially when comparing 24-month-old and 6-month-old mice (Figure 2A). The pattern was confirmed by the quantification of the vimentin total signal intensity, which was significantly increased in 12-, 18- and 24-month-old mice when compared to the control (Figure 2B).

In retinal sections of 6-month-old mice, it was possible to visualize a very scarce number of GFAP-positive cells (Figure 2A). As age advanced, GFAP immunoreactivity increased and appeared in several layers of the retina. Specifically, in 24-month-old animals, GFAP immunoreactivity was present throughout the INL (Figure 2A, indicated by a white arrow at 24 months old). At this layer, GFAP labeling was associated with activation on Müller cells, while GFAP-positive cells close to the RGC layer corresponded to reactive astrocytes. The quantification of the total signal intensity showed a statistically significant increase in GFAP levels, indicating reactive gliosis at older ages (Figure 2C).

Microglial reactivity was also analyzed using the Iba1 marker [58]. In 24-month-old mice, Iba1-positive cells clearly exhibited a reactive morphology and were found throughout both retinal plexiform layers (Figure 2A). A statistically significant increase in Iba1-positive cells was confirmed by quantification in the 24-month-old mice when compared to the 6-, 12- and 18-month-old mice (Figure 2D).

Altogether, these data indicate that features of glial reactivity are increased in the naturally aging mice model used in this study, as expected [4].

### 3.3. Synuclein Distribution in the Neural Retina of Naturally Aged Mice

Having confirmed that clear age-related features of degeneration and inflammation were present in our naturally aging mice model, we aimed to assess the synuclein distribution profile along the retinal layers and to verify whether there was a pattern of changes with aging. Each of the synuclein proteins follows a specific arrangement of localization in the retina. It has already been described by a few studies that aSyn and bSyn are distributed in a more similar way, being found to colocalize mainly in the IPL and in individual cells in the INL [33,34,39,59].

**Figure 3:**
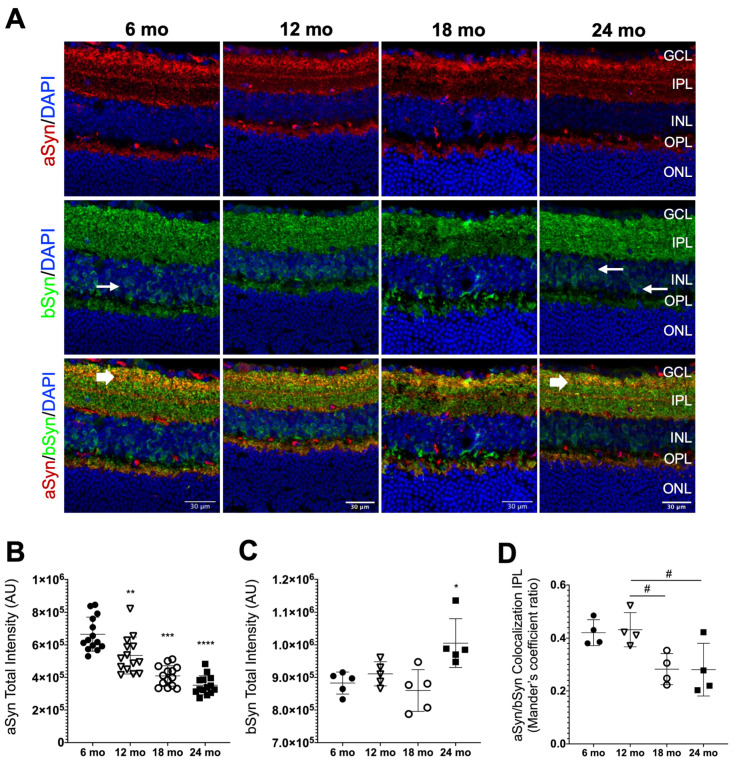
aSyn and bSyn distribution profiles in mice retinas. (**A**) Representative immunofluorescence of aSyn (red) and bSyn (green) merged with DAPI at the indicated ages. The thin white arrows indicate bSyn expression around cell bodies in the INL at 12 and 24 months old (mo). Thick white arrows show colocalization sites between aSyn and bSyn in the IPL and OPL at 6 and 24 mo. (**B**) Quantification of aSyn fluorescence intensity throughout the retina (*n* = 14). (**C**) Quantification of bSyn fluorescence intensity throughout the retina (*n* = 5). (**D**) Quantification of aSyn and bSyn colocalization in the IPL (*n* = 4). Scale bar = 30 mm. Fluorescence intensity analysis was performed using approximately 4 representative images of the central region of the retina from each animal per age group. Values are represented by mean ± SD. Mice with 12 (∇), 18 (○) and 24 mo (■) were compared to 6 mo (●) mice using an ordinary one-way ANOVA multiple comparison test (* *p* < 0.05, ** *p* < 0.01, *** *p* < 0.001 and **** *p* < 0.0001). Mice with 18 and 24 mo were compared to 12 mo mice using an ordinary one-way ANOVA multiple comparison test (^#^ *p* < 0.05).

We also observed that aSyn was mainly present in the IPL, with a marked distribution in the different strata of this layer, as shown by intense immunolabeling in confocal microscopy images of IHC assays (Figure 3A). This is consistent with studies in vertebrates that show its presence at presynaptic endings of bipolar and amacrine cells, pointing to its involvement in the modulation of neurotransmission [34]. Although an aSyn signal was also observed in the OPL (Figure 3A), further analysis using a different secondary antibody led us to realize that aSyn localization in this layer was questionable (Supplementary Figure S2), in agreement with previous studies [34,59]. Regarding bSyn, its distribution in the IPL was more homogeneous than aSyn, with no apparent location in specific strata. Additionally, bSyn had a different pattern of distribution from aSyn and it appeared to surround the cellular bodies of neurons in the INL, which could indicate functional implications, apart from presynaptic transmission (Figure 3A). bSyn was also present in the OPL, showing strong immunolabeling (Figure 3A). Interestingly, bSyn became widely present at the INL as the animals become older, easily noticeable when comparing 6-month-old mice with 24-month-old mice (Figure 3A; indicated by small arrows).

To evaluate the effect that the aging process had on the aSyn and bSyn distribution in the retina, we measured the intensity of immunolabeling of those two proteins, as well as their colocalization in mice retinal tissue of different ages. Quantifications revealed that aSyn levels tended to decrease with aging (Figure 3B), while the bSyn levels were higher in 24-month-old mice when compared with the control (Figure 3C). However, no age-related alterations were observed in the aSyn and bSyn protein levels (results not shown). Exploring the spatial resolution that IHC allows, we evaluated aSyn and bSyn immunolabeling intensity in the different layers. However, no significant differences were observed (supplementary Figure S3).

Furthermore, it is essential to understand how aging can interfere with the aSyn and bSyn colocalization pattern in the same retinal cell sub-populations, as reports point to a synergistic role in physiological and pathological conditions [7,16,20,21,22,24,30,31,32]. aSyn and bSyn seemed to colocalize more throughout the IPL in younger mice than in the retinas of older animals (Figure 3A; large arrows). Manders’ colocalization coefficient showed that in the IPL, aSyn and bSyn colocalization decreased in 18- and 24-month-old mice, compared with 12- and 6-month-old mice (Figure 3D).

**Figure 4:**
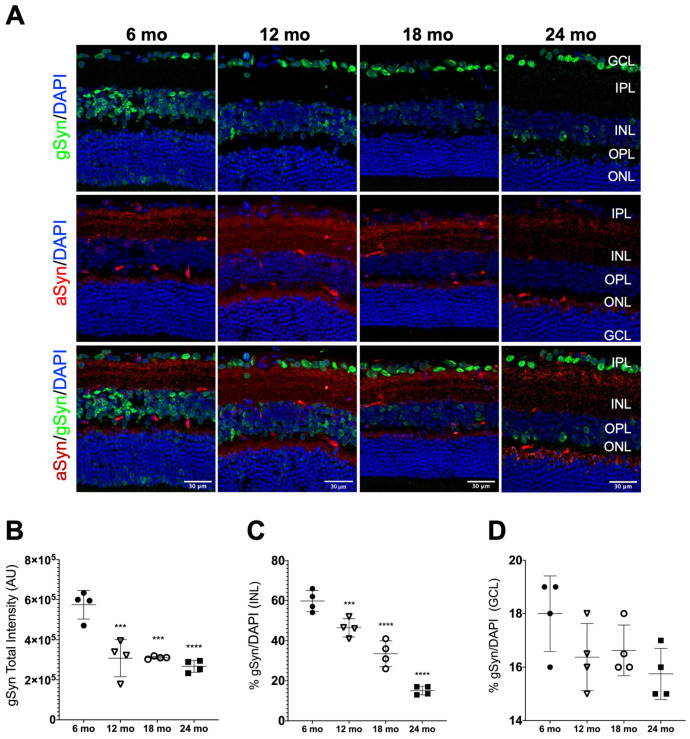
gSyn distribution profile in mice retinas. (**A**) Representative immunofluorescence of gSyn (green) and aSyn (red) merged with nuclear DAPI staining of retinal sections at the indicated ages. (**B**) Quantification of gSyn fluorescence intensity in all retinal layers (*n* = 4). (**C**) The gSyn-positive cell body count in the GCL and (**D**) in the INL (*n* = 4). Scale bar = 30 μm. Fluorescence intensity analysis was performed using approximately 4 representative images of the central region of the retina from each animal per age group. Values are represented by mean ± SD. Mice of 12 (∇), 18 (○) and 24 months of age (mo) (■) were compared to 6 mo (●) mice using an ordinary one-way ANOVA multiple comparison test (*** *p* < 0.001 and **** *p* < 0.0001).

We also evaluated the distribution profile of gSyn in the mice retinas and its relationship with aSyn. Unlike aSyn and bSyn, which showed a main localization in synaptic layers, gSyn was visibly mainly localized in the cell bodies, in the GCL and INL, with its presence becoming visibly fainter in the INL with aging (Figure 4A). To confirm the pattern seen in the images, we counted the number of cells positive for gSyn in the GCL and INL. The quantification result confirmed the decrease in gSyn total intensity levels with aging (Figure 4B). This decrease was mainly due to reduced levels of gSyn in the INL with aging (Figure 4C), while in the GCL, no significant differences in the gSyn positive cell body count were found (Figure 4D).

Considering the colocalization of gSyn with aSyn, we did not visualize it in any of the layers of the retinas analyzed (Figure 4A). Additionally, the quantification of Manders’ colocalization coefficient showed values of zero or close to zero for all measurements. Due to this, we postulated that these two proteins were not present in the same cell sub-population of the mice retina.

### 3.4. aSyn Distribution in Specific Retinal Neuronal Populations in Naturally Aged Mice

We then performed a more detailed characterization of the aSyn distribution with specific markers for different retinal populations with aging.

**Figure 5:**
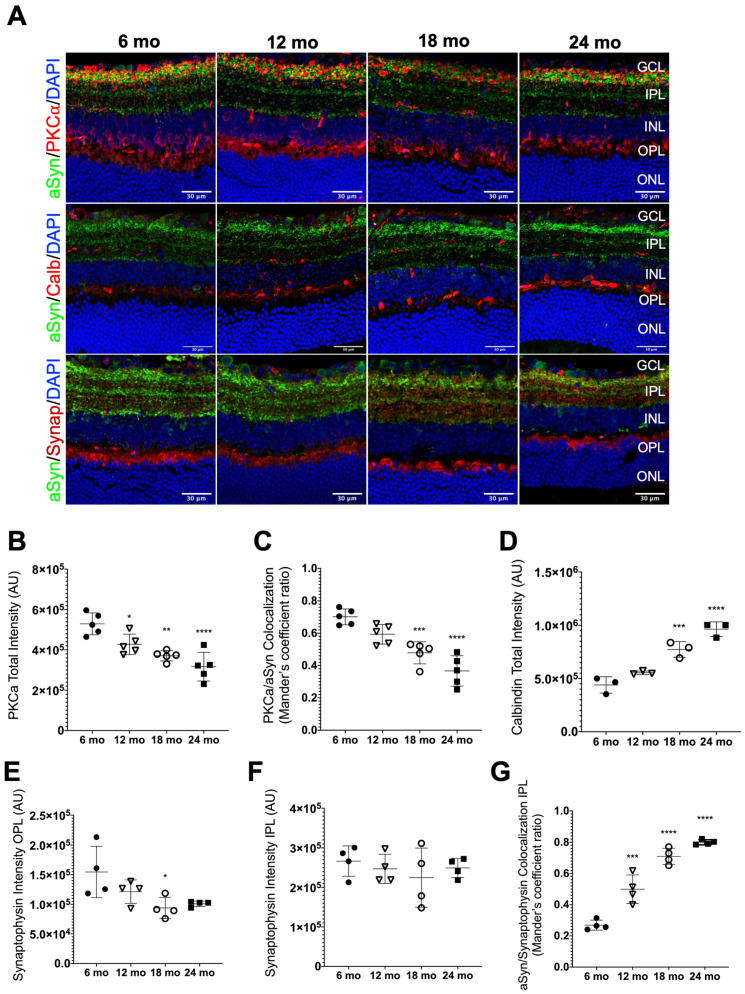
Characterization of aSyn distribution with different retinal cells. (**A**) Representative immunofluorescence of aSyn (green) and PKCα (red), calbindin (red) and synaptophysin (red), respectively, of retinal sections at the indicated ages. (**B**) Quantification of PKCα fluorescence intensity in all retinal layers (*n* = 5). (**C**) Quantification of aSyn and PKCα (bipolar cells) colocalization in the GCL, IPL, INL and OPL (*n* = 5). (**D**) Quantification of Calbindin (horizontal cells) fluorescence intensity in all retinal layers (*n* = 3). (**E**) Quantification of Synaptophysin fluorescence intensity in the OPL and (**F**) in the IPL (*n* = 4). (**G**) Quantification of aSyn and Synaptophysin colocalization signals in the IPL (*n* = 4). Scale bar = 30 μm. Fluorescence intensity analysis was performed using approximately 4 representative images of the central region of the retina from each animal per age group. Values are represented by mean ± SD. Mice of 12 (∇), 18 (○) and 24 months-old (mo) (■) were compared to 6 mo (●) mice using an ordinary one-way ANOVA multiple comparison test (* *p* < 0.05, ** *p* < 0.01; *** *p* < 0.001 and **** *p* < 0.0001).

PKCα has been shown to be specifically expressed in rod bipolar cells [60], where it is localized in the cell body, dendrites, axons and in the synaptic terminals [61]. Therefore, we aimed to evaluate how rod bipolar cells behaved in aging retinas by the distribution profile of PKCα intensity and to establish a correlation with the expression of aSyn, particularly in the IPL. As expected, PKCα was specifically localized, delimitating the cell bodies of bipolar cells in the INL, as well as in all its extension in the IPL, with high intensity of signal in the synaptic terminals adjacent to the GCL, in strata S5 (Figure 5A), as previously described [62]. In the retinal sections of 6-month-old mice, the immunolabeling was more intense, especially in the INL and IPL, than in the other ages (Figure 5A). Indeed, we measured the total intensity of PKCα in all layers that this protein seemed to be present, and we confirmed the significant decrease with aging, as represented by the confocal images. (Figure 5B).

Regarding aSyn colocalization with PKCα, we observed a decrease in the colocalization between these proteins when comparing 18- and 24-month-old mice with 6-month-old mice (Figure 5C). However, it is important to keep in mind that the total intensity of the aSyn signal also declines as the retina becomes older (Figure 3B) and that rod bipolar cells are functionally affected in age-related conditions [63].

Calbindin is a calcium-binding protein involved in calcium metabolism and transport, widely distributed in the nervous system, and expressed in specific populations of neurons. In the retina, calbindin is a marker of horizontal cells, but to a lower degree, it also labels some amacrines [53].

We observed that the immunolabelling of calbindin was more predominant in the basal region of the INL, close to the OPL, characterizing the horizontal cell population. It was also possible to visualize calbindin staining on the apical part of the INL and GCL, where amacrine cells are located, but to a lesser extent (Figure 5A). With aging, we observed that the calbindin signal is becoming more intense, especially in the INL/OPL (Figure 5A). This fact was confirmed by calbindin total intensity quantification, where a significative increase of the signal intensity of 18- and 24-month-old retinas was seen, when compared with the control animals (Figure 5D). No colocalization was observed between calbindin and aSyn, indicating that aSyn was not present in horizontal cells of mice retinas.

Synaptophysin is an abundant synaptic vesicle glycoprotein known to participate in neurotransmitter release, vesicle docking and recycling, in association with other proteins [64]. In the retina, synaptophysin is expressed in photoreceptor axon endings in the OPL and in bipolar and amacrine cell terminals in the IPL [65,66] and was previously shown to colocalize with aSyn in presynaptic terminals of the retina [34]. Here, we analyzed it to evaluate how the aging process interferes with synapses. We observed that synaptophysin immunostaining is distributed throughout the synapse-rich IPL and OPL of the retina, as expected [65,66], and that synaptophysin colocalizes with aSyn at presynaptic terminals in the IPL (Figure 5A), as reported before [34]. Then, we quantified the intensity of the synaptophysin signal in the two plexiform layers. We observed significant changes in synaptophysin immunoreactivity with a tendency to decrease in the OPL with age, even if it is only significantly lower in 18-month-old mice when compared to the control (Figure 5E). The synaptophysin signal intensity remains unaltered in the IPL with aging (Figure 5F). These data suggest that the decrease in synaptophysin protein levels and/or increase in its degradation may begin to occur in the OPL, which could affect the synapses made by horizontal cells and photoreceptor cells.

Next, we performed intensity quantification to address the effect of natural aging in the colocalization pattern of aSyn and synaptophysin in the IPL. For this, we considered the Manders’ colocalization coefficient. We found that the colocalization between both proteins increased abruptly with aging when comparing 12-, 18- and 24-month-old mice retinas with controls (Figure 5G), which could suggest alterations in synaptic vesicles dynamics in the IPL with aging linked with aSyn physiological function.

## 4. Discussion

The physiological role of synucleins in neuroretina is very poorly characterized. However, it is evident that they are critical for presynaptic function [67] and are important for visual function in aging, as triple synuclein KO mice display age-dependent retinal dysfunction and blindness [28]. Understanding the mechanisms of retinal aging is essential for understanding age-related diseases and identifying targets for therapeutic intervention.

Some studies have pointed out that synucleins can also acquire a toxic role in the retina. Namely, markers of aggregated aSyn have been found in the retinas of PD patients and DLB patients [38,39,40]; gSyn inclusions have also been observed in retina AD patients [33] and in aged degenerating retinas with glaucoma [43]. Aggregation of aSyn and acquisition of new toxic functions are intimately linked to its loss of function, as less protein will be available to play its normal role in the synapses.

Here, we characterized the retinal synuclein profile over aging, using naturally aging animals from 6 to 24 months old. Although genetic models can be powerful tools for dissecting synuclein molecular mechanisms, it is becoming clear that they present important limitations. Namely, they show aSyn expression in specific retinal cells where naturally the protein is not being expressed and depending on the promoter used [33,47,48]. In addition, the use of accelerated aging models has some constraints, as they are more suitable models for studying specific molecular pathways involved in aging [3]. Therefore, we decided to use naturally aged WT mice from 6 to 24 months old. In mice, a 6-month-old is considered to be late adulthood; between 10- and 15-months of age, senescent changes start to occur, even if the biomarkers of aging are still not detected [68]. At 15 months old, considered the upper limit for middle age, mice reproductive functions cease. Then, from 18 months of age on, the post-senescence phase is characterized by detectable old age biomarkers [68].

We started by characterizing the alterations suffered in the retina of our mouse strain C57BL/6J to validate it and compare it to what was previously described. Changes in the thickness of the nuclear and plexiform layers of the retina are predicted to occur in aging animals [53,69]. The thinning of the retina is not a matter of volume change since the total volume of the retina remains unaltered [70]. In our natural aging mice model, followed up to 24 months old, we confirmed a significant decrease in the ONL area size in all ages compared to 6-month-old mice and in IPL when comparing 24-month-old mice to the control. These results are in accordance with previous studies in rats that relate thinning of the INL and ONL to a larger extent and the plexiform layers, with IPL being more affected than OPL [54,69,70]. The changes observed in the nuclear layers could be explained either by alterations in cell density or by loss of cellular bodies, characteristic of retinal degeneration. The significant decrease in the IPL in 24-month-old mice can indicate a lower number of synapses and, therefore, reduced functionality. Regarding the plexiform layers, mislocalization and decreased density of synapses were observed in aging mouse and human retinas [71,72]. This phenomenon can be anticipated by cell loss in the INL and GCL. In fact, we observed a significant decrease in the number of DAPI-positive cells in the ONL with aging, as well as a decrease in OS layer thickness, pointing to a degenerative process. This pattern agrees with previous studies that related degeneration of photoreceptor cells to aging of the retina [50,72]. Analyses of gene expression changes in the rod photoreceptor cells of a transgenic mouse also reveal changes in pathways associated with aging in older animals, which can lead to cell death [73].

Photoreceptor cells, especially rods, are the predominant cell type in the retinas of mammals and have already proven to be the most vulnerable to the effects of aging, leading to an important decline in rod-mediated visual function. In *retinitis pigmentosa* and age-related macular degeneration, the loss of rods and the functional decline of rod-mediated vision come before the beginning of cone degeneration, since rod cells are responsible for releasing trophic factors that contribute to cone survival [73]. We also observed an OS layer decrease with age, as well as morphological alterations of the photoreceptor disks and decreased levels of rhodopsin. The observed decrease in ONL thickness can indicate a possible degenerative profile of photoreceptor cells triggered by the aging process.

The process of aging in mammalian retinas is very complex, with several molecular pathways becoming activated, leading to tissue dysfunction and posterior vision loss [49,50,51]. The progressive loss of RGCs and changes in the optic nerve are characteristics of a very well-known age-related retinal disease, glaucoma. Even though the molecular processes behind RGC damage are still poorly understood, they are currently being extensively studied. RGCs, together with rod photoreceptors, are prone to loss during aging [74]. Age-related loss of neurons in the inner retina has been well established in past years. Several histological studies of RGC somas, as well as the number of axons in the nerve fiber layer (RNFL), have been investigated since one of the aging effects in the retina is the thinning of the RNFL [75]. In this natural aging mouse model, we also observed a decline in the number of Brn3a-positive RGCs, particularly in 18- and 24-month-old mice, which suggests a possible cell degeneration process followed by cell death. This methodology was used because the GCL is also populated by some displaced amacrine cells [76], making it impossible to predict RGC loss without a specific marker (Brn3a).

During the progression of retinal degeneration, the microenvironment of the retina suffers important changes that can interfere with retinal physiology. Müller cells undergo a process of activation, also known as gliosis, which is characterized mainly by an overexpression of intermediate filament proteins, such as GFAP and vimentin. The increase of those proteins can culminate in the formation of scar tissue that surrounds the retina and hypertrophy of Müller glia cells, showing progressive growth in the proliferation of fibrous processes. Moreover, Müller cells have been associated, in comparison with astrocytes in the brain and spinal cord, with a retinal wound-healing response after injury and are important players in the regenerating processes [77]. Our data also showed an induction of glial reactivity with natural aging, involving not only Müller cells but also astrocytes and microglia.

There are at least 10 different types of bipolar cells, 50 morphologically different amacrine cells and 10 to 15 morphological types of ganglion cells [78]. The IPL is where amacrine and bipolar interneurons establish synapses with ganglion cells. Here, both the bipolar cell axon terminals and the ganglion cell dendrites stratify at different levels. Our data and data obtained from others [33], suggest that in general, aSyn and bSyn are expressed in different retinal interneurons, with aSyn mainly localized in presynaptic terminals that end in different strata, while bSyn shows a broader presynaptic distribution. However, in some of these presynaptic terminals, aSyn and bSyn colocalize, indicating that they are co-expressed in some specific interneurons.

While the aSyn protein levels did not change significatively in the retina with aging (results not shown), its immunofluorescence signal was found to significantly decrease with aging, based on immunofluorescence quantification of retina sections. The homogenization of the retina tissue and total levels assessment can lead to a dilution effect of the alterations that occur in specific cell sub-types. Moreover, pS129 aSyn was not detectable either by western blot or IHC in the tested conditions (data not shown). In the aging human brain, the levels of aSyn mRNA decrease while, depending on the region, its protein levels do not change or even increase in the substantia nigra [79], reinforcing the idea that the protein is being stabilized by post-translation modifications [79] or its degradation mechanisms are less effective [3,5]. aSyn-decreased levels in the presynaptic terminals of retinal interneurons can have a relevant impact on their synaptic function. Even in those interneurons where bSyn is being co-expressed, the aSyn function in vesicle clustering and SNARE-complex assembly cannot be replaced by bSyn [80]. We hypothesized that the reduction of aSyn in the IPL could result in compensatory mechanisms, such as alterations in SNARE proteins or through cysteine string protein alpha (CSPa), also involved in synaptic SNARE complex formation acting synergistically with aSyn [81]. However, observations performed in triple synuclein KO mice showed age-dependent alterations in the synaptic SNARE proteins SNAP-25 and synaptobrevin-2 [28]. Although these and other possible mechanisms could be enough to compensate for aSyn loss of function in triple synuclein KO young mice, they become insufficient in old mice, where age-dependent retinal dysfunction and blindness were observed [28].

Regarding bSyn, we observed that although its protein levels did not change significatively in the retina with aging (results not shown), its immunofluorescence signal significantly increases in retina sections of 24-month-old mice. bSyn is not involved in the SNARE-complex assembly but was recently described as potentiating synaptic vesicle dopamine uptake in the absence of other synucleins through a mechanism not completely clarified [82]. In the retina, the only dopaminergic cells are a subpopulation of amacrine cells [83]. Our results show that bSyn expression is broader, so we can envisage that other physiological roles can be taking place by bSyn that need future studies using single, double and triple synuclein KO mice.

Interestingly, aSyn and bSyn colocalization in some presynaptic terminals in the IPL decreased with aging. According to recent experimental evidence, aSyn and bSyn are known to have the capability to interact with lipid bilayer membranes and aSyn has a higher affinity to that binding than bSyn, which makes the first protein more prone to form lipid-induced amyloid formation than the latter [84,85]. These studies also determined that bSyn decreases the rate of aSyn amyloid formation, being inhibitory in the presence of lipid membranes and conferring neuroprotection to the cells. The mechanisms of inhibition remain unclear. Some authors propose that the two proteins interact with each other directly [86,87] and others say that the interaction between aSyn and bSyn is rather a competition for binding sites on surfaces [85].

Very little is known about the mechanisms behind aSyn and bSyn colocalization profiles, as well as their implications for the functionality of the cell types in the retina. A recent study using C57BL/6 mice also showed that bSyn has a direct effect on the physiological function of aSyn in synaptic vesicles. When these proteins colocalize and there is an excess amount of bSyn, aSyn interacts less with the synaptic vesicles due to competition, reducing aSyn affinity for synaptic vesicles and causing functional changes to neuronal synapses [80]. Given that, our results suggest that there might be an imbalance of synucleins, especially in the IPL, that influence the correct pattern of colocalization between those proteins and possibly affect neuronal function and plasticity.

Previous studies described that gSyn could be considered a specific marker for retinal ganglion cells and that loss of ganglion cells is associated with downregulation of gSyn gene expression [88]. Here, we did not observe significant alterations in the number of gSyn-positive ganglion cells with aging. However, when this population was analyzed based on the Brn3a marker, a significant decrease in the number of Brn3a-positive cells was observed in the 18- and 24-month-old retinas. It is possible to hypothesize that not all retinal ganglion cells express gSyn, as other RGC subtypes are positive for Brn3a. The gSyn physiological function is far to be clarified, but it is known that gSyn is not involved in the SNARE-complex assembly [22], and has reduced presynaptic localization, in agreement with its localization in ganglion cell bodies and in the INL. Its role as a modulator of aSyn synaptic function is excluded in the retina [80], as no colocalization was observed between gSyn and aSyn.

The presence of gSyn in the INL of retinal tissue has not yet been reported. Further analyses are necessary to investigate which cells in the INL have gSyn expression and the implications of the decrease observed with aging.

Lastly, the colocalization of aSyn and synaptophysin (synaptic vesicle marker) increases with aging, indicating the involvement of aSyn at the synapses mainly in the IPL. This interplay is especially affected by aging. This finding is also in agreement with other studies on the aged nervous system [27,89]. No evidence is available suggesting that synaptophysin can replace or be a neuroprotector from aSyn loss of function. In triple synuclein KO mice, this is one of the proteins that is not significantly altered in either young or old mice [28]. However, we can hypothesize that a reduction in aSyn function with aging can result in problems in synaptic vesicles docking and fusion with the plasma membrane, leading to the accumulation of synaptic vesicles in the presynaptic terminals.

Overall, this study allowed us to demonstrate that synuclein distribution in mouse retinas is altered with aging. Our results strongly suggest that natural aging can be responsible for important changes regarding neuroinflammation and degeneration processes, but more importantly, for structural and molecular changes that could affect synuclein family function.

In future studies, it will be important to explore both the physiological and pathological roles of the different synuclein family members in the retina, as the main available studies are focused on the brain. For the physiological role, single, double and triple synuclein KO mice can be very useful to correlate molecular and cellular alterations in the retina with visual function analysis, such as electrophysiological and behavioral assessments. On the other hand, little is known about aSyn’s pathological role in the retina and its relationship with brain pathology. Namely, pathological alterations of aSyn in the retina can represent a window to the brain and can be explored as a biomarker for the diagnosis and monitoring of disease progression [39]. Alternatively, aSyn pathology in the eye could represent a new body-to-brain PD propagation route. It will be important to explore these possibilities using appropriate PD animal models, such as the aSyn preformed fibril (PFF) model [90], which allows the injection of PFF in different locations (p.e., intravitreal) inducing endogenous aSyn aggregation in a prion-like mechanism in WT mice of different ages. Moreover, it will be interesting to evaluate if the alterations here reported to occur in synuclein distribution with aging are conserved in human eyes obtained from both healthy and PD donors.

## Data Availability

The data that support the findings of this study are available from the corresponding author upon reasonable request.

## Supplementary Materials

The following supporting information can be downloaded at: https://www.mdpi.com/article/10.3390/biom13010180/s1, Figure S1: Evaluation of the thickness or cell loss of the nuclear layers, ganglion cell layer and photoreceptor layer of mice retinal sections at indicated ages. Figure S2: Immunofluorescence of the two aSynuclein (aSyn) antibodies used in this study. Figure S3: RNA expression levels of *Snca*, *Sncb* and *Sncg* in mice retinas at the indicated ages.
