# Supplementary figures and images for: Age-Related Changes of the Synucleins Profile in the Mouse Retina
Source: Biomolecules. 2023 Jan 15;13(1):180. doi: 10.3390/biom13010180 (PMC9855780; doi:10.3390/biom13010180)

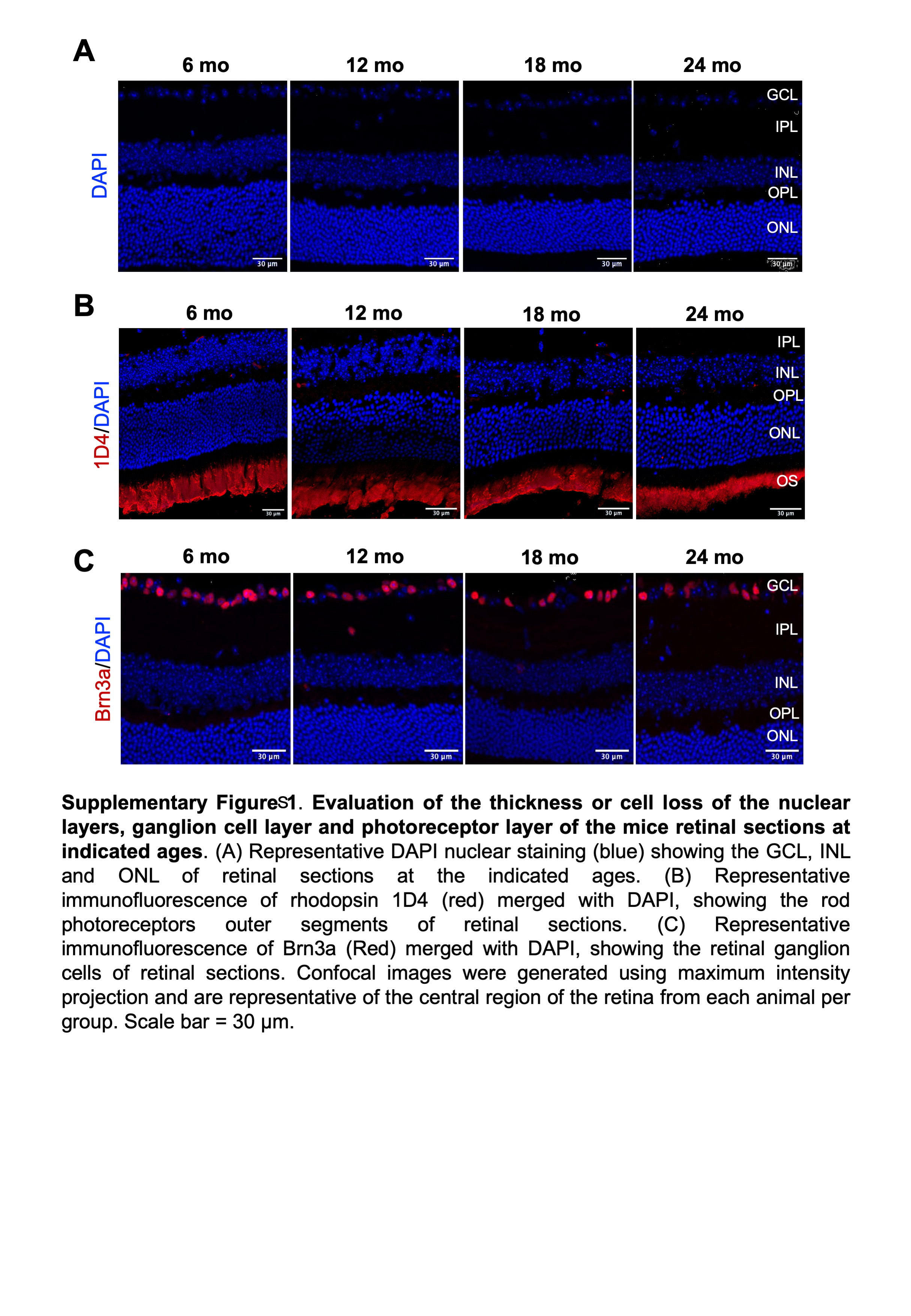

Supplement: Supplementary file 1 [file biomolecules-13-00180-s001.zip › Supp_Figure_S1.tif]

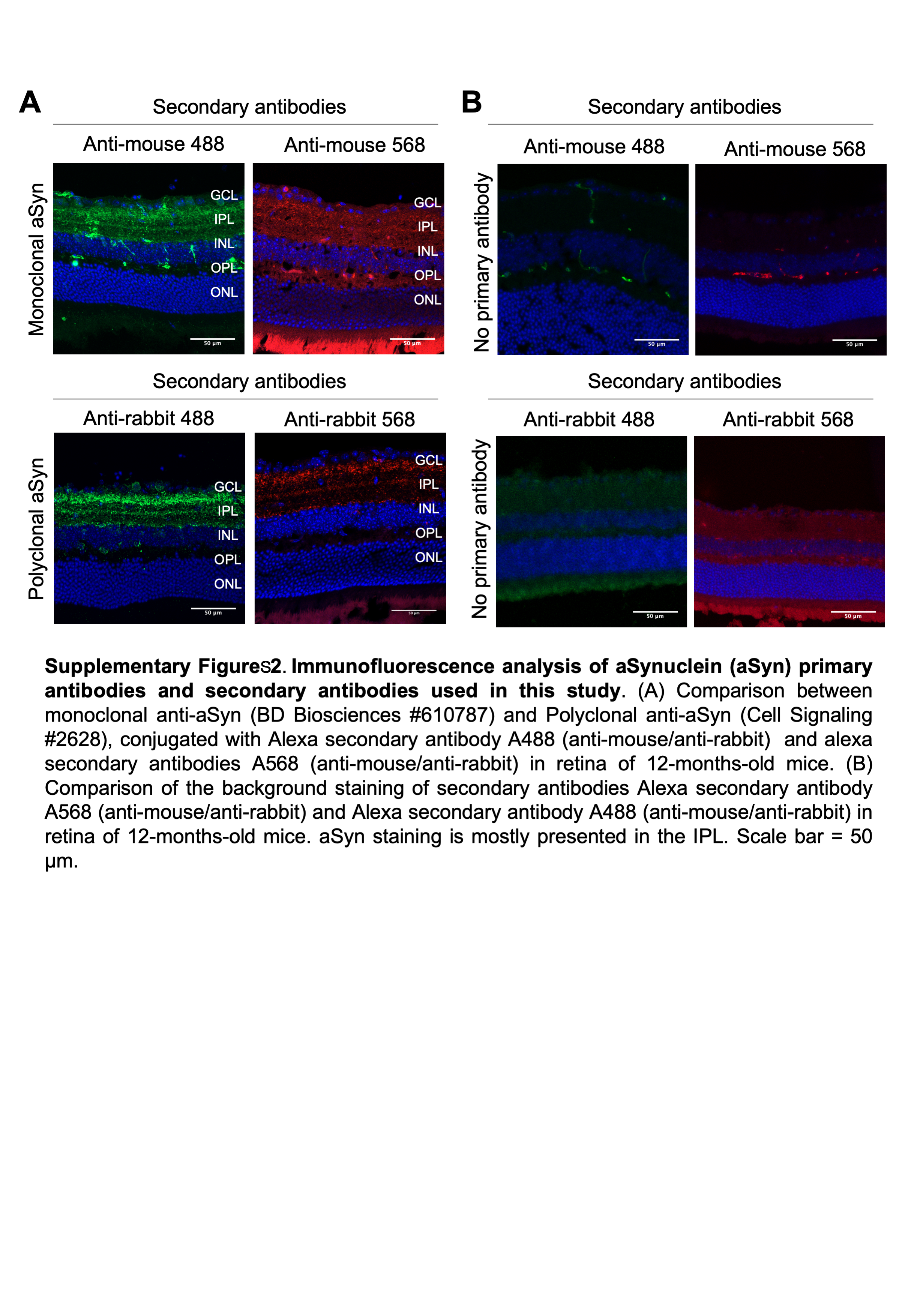

Supplement: Supplementary file 1 [file biomolecules-13-00180-s001.zip › Supp_Figure_S2.tif]

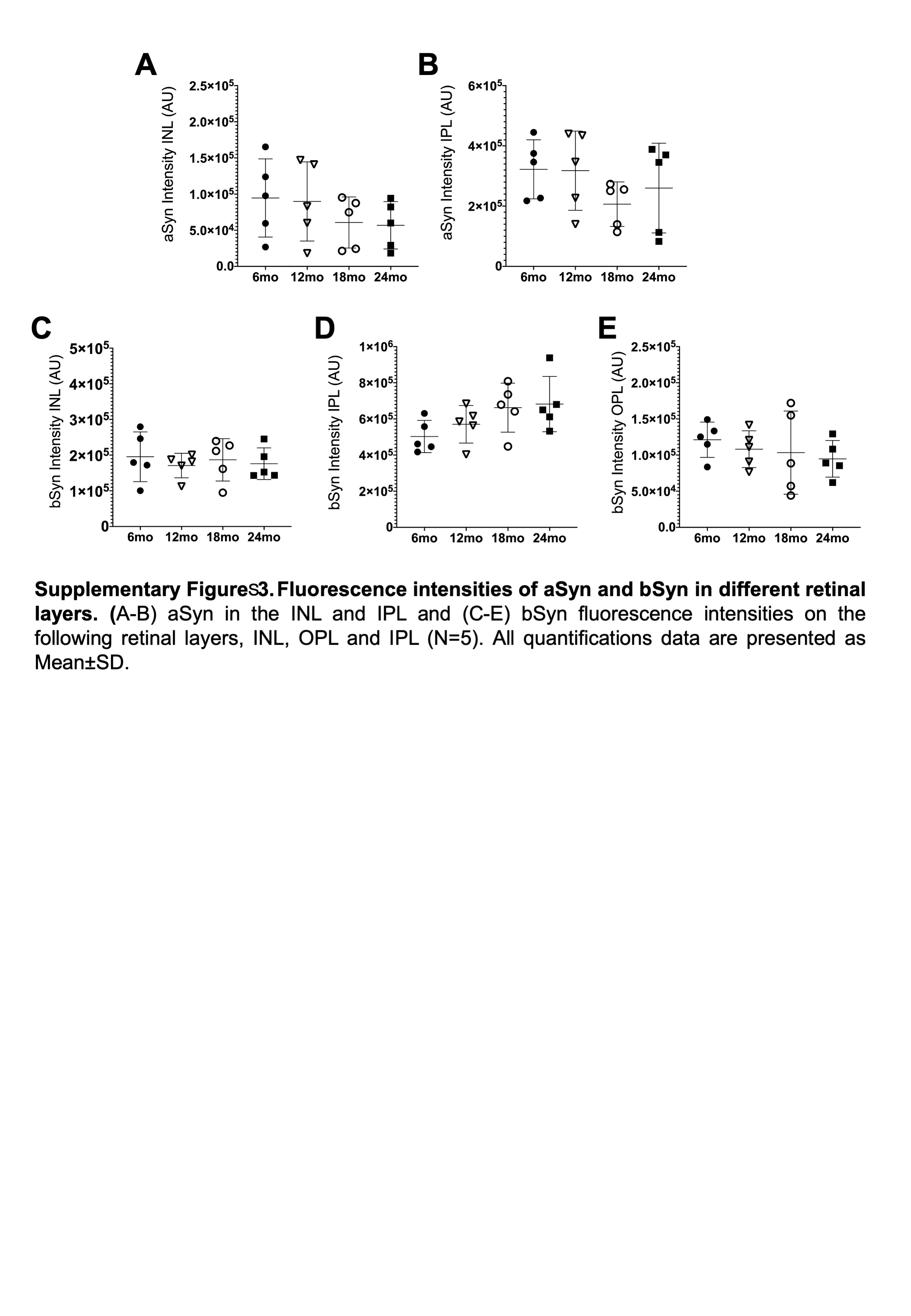

Supplement: Supplementary file 1 [file biomolecules-13-00180-s001.zip › Supp_Figure_S3.tif]
